# A Systematic Review of 3D-Printing in Spine Surgery: Applications, Accuracy, and Outcomes

**DOI:** 10.3390/healthcare14193344

**Published:** 2026-10-08

**Authors:** Majdi Hashem

**Affiliations:** Department of Surgery, Faculty of Medicine, Imam Mohammad Ibn Saud Islamic University (IMSIU), Riyadh 11623, Saudi Arabia; mahashem@imamu.edu.sa; Tel.: +966-507739919

**Keywords:** 3D-printing, outcomes, precision, spine surgery

## Abstract

**Background**: Three-dimensional (3D)-printing has increasingly been applied in spine surgery for patient-specific planning, instrumentation, and implants. This systematic review and meta-analysis aimed to evaluate the applications, technical accuracy, and outcomes of 3D-printing in spine surgery. **Methods**: This systematic review study was conducted according to the Preferred Reporting Items for Systematic Reviews and Meta-Analyses (PRISMA). A comprehensive literature search was conducted for the available literature that was published before 7 November 2024, using three medical databases (PubMed, Scopus, and Cochrane). **Results**: A total of 40 studies were included in this systematic review, of which 28 (70.0%) were retrospective design studies. Seven studies were included in the meta-analysis of screw placement accuracy, which showed a significantly higher probability of achieving the defined accuracy outcome with 3D-printing-assisted techniques (RR = 1.12, 95% CI: 1.06–1.19; *p* = 0.003). Meta-analysis of eight studies showed significantly shorter surgical time (MD = −28.97 min, 95% CI: −43.41 to −14.53; *p* < 0.001) and lower blood loss (MD = −80.70 mL, 95% CI: −149.92 to −11.48; *p* = 0.022) with 3D-printing-assisted approaches. However, substantial heterogeneity was observed for surgical time and blood loss. **Conclusions**: The available evidence suggests that 3D-printing-assisted approaches may improve technical accuracy and reduce surgical time and blood loss in spine surgery. However, the findings should be interpreted cautiously because of substantial clinical and methodological heterogeneity and limitations in the quality of the available evidence. Further well-designed prospective studies and randomized controlled trials are needed to confirm these findings.

## 1. Introduction

Three-dimensional (3D)-printing, also known as solid free-form technology, additive manufacturing, or rapid prototyping [1,2,3], is an advanced technology that uses three-dimensional (3D) computer software to produce individualized 3D physical models [2,4,5,6]. Since the late 1990s and early 2000s, 3D technology has become more prevalent in multiple domains, such as industrialization, healthcare, and aviation [7]. The use of 3DP in healthcare includes medical education, drug testing, hearing aids, surgical planning, and dental models [8,9,10]. Neurosurgery, especially spine surgery, is one medicine area where 3DP technology delivers significant potential advantages [11].

The applications of 3D-printing in spine surgery that have proven to offer significant benefits include its use as an educational tool for training and patient–physician communication, anatomical visualization, preoperative planning, surgical templates, and custom prosthetic design [10,12,13,14,15,16,17,18,19,20,21,22,23,24,25,26,27,28,29,30,31,32]. In addition, the benefits of using 3D-printing bio-models in spine surgeries include enhancing patient outcomes, enabling individualized treatments, making surgery more visible, reducing surgery time, raising the safety and accuracy of the surgical procedure, and improving implant properties. However, several major challenges, including a long learning curve, significant time requirements, and high costs associated with 3D-printing techniques, vary depending on the technology used [2,8,11,33,34,35,36].

Any technique that may improve the accuracy of spine surgeries and assist in surgical planning can enhance patient results because of the delicate nature of the structures surrounding the spine and its intricate anatomy [37]. Thus, the clinical applications and research of 3D-printing technology in spine surgery have increased significantly in recent years [38]. Due to the rapid development of 3D-printing technology and its increasing use in surgical applications of the spine, there is a constant need to provide the latest evidence regarding its use, accuracy, and results to help achieve optimal surgical results and improve patients’ outcomes, health, and quality of life. Therefore, this systematic review aims to determine the applications, precision, and outcomes of 3D-printing in spine surgery.

## 2. Methods

This systematic review study was conducted according to the Preferred Reporting Items for Systematic Reviews and Meta-Analyses (PRISMA 2020). No review protocol as prospectively registered for this systematic review. The PRISMA 2020 statement takes into account the combination of randomized and non-randomized evidence. PRISMA 2020 offered the most thorough and widely accepted framework as the evidence it incorporated included both observational studies and randomized controlled trials. The Cochrane RoB 2 tool for randomized trials and the modified Newcastle–Ottawa Scale for non-randomized studies were the study-design-specific instruments used to evaluate methodological quality independently. A comprehensive literature search was conducted for the available literature that were published before 7 November 2024, using three medical databases (PubMed, Scopus, and Cochrane). Titles and abstracts were screed according to pre-established inclusion and exclusion criteria, followed by full text assessment of potentially eligible studies. Study selection was performed by the author. The search strategy combined terms related to three-dimensional printing and spine surgery, including controlled vocabulary and free-text terms. The search terms included: ((“3D Printing”[MeSH] OR “”3D-printing” OR “Three-Dimensional Printing”) AND (“Spine Surgery”[MeSH] OR “spinal surgery” OR “spine procedures” OR “spinal procedures” OR “spinal deformity” OR “spinal fusion” OR “vertebrae surgery”)) AND (applications OR accuracy OR precision OR reproducibility OR outcomes OR “clinical outcomes” OR efficacy OR effectiveness or benefits). The same conceptual search strategy was applied across the three databases, with database-specific syntax adapted as required. Titles, abstracts, and full text studies were reviewed according to pre-established inclusion criteria. Studies were eligible if they were original human studies evaluating the use of three-dimensional (3D) printing in spine surgery, including patient-specific models, surgical guides, instrumentations, implants, or other 3D-printing-assisted applications. Studies were required to report at least one relevant technical or clinical outcome, such as instrumentations or screw placement accuracy, surgical time, blood loss, complications, or other surgical or patient-related outcomes. Both comparative and non-comparative study design were eligible. No restriction was applied on study design, gender, age, or type of spine surgery. Only studies published in English were included. Reasons for exclusion were recorded during the full text assessment, and the study selection process was summarized in the PRISMA flow diagram.

### 2.1. Data Extraction

The main outcome of interest in this systematic review was to determine the applications, precision, and outcomes of 3D-printing in spine surgery. For this review, accuracy was interpreted as the closeness of the achieved surgical results to the intended or planned position, particularly for instrumentation and screw replacement. Precision was interpreted as the consistency or reproducibility of the achieved surgical results across measurement or procedures. Because the terminology and outcome definitions varied across the included studies, reported measures such as screw placement accuracy, deviation from the planned trajectory or position, and other measures of technical performance were extracted according to the definitions used by each study. Accuracy and precision were therefore considered related but distinct concepts and were not assumed to be interchangeable. Data were extracted by the author using a standardized data extraction approach. The extracted information included study design, aim, sample size, male to female ratio ad frequencies, mean age of the study participants, application of 3D-printing in the spine surgery, and reported precision and clinical outcomes. Data included in the meta-analysis were also excluded by the author including the blood loss in ml, and surgical time in minutes.

### 2.2. Statistical Analysis

Descriptive statistics were used to summarize study characteristics and outcomes that could not be quantitatively pooled because of differences in outcome definitions, reporting methods, or the limited number of studies. Where sufficient comparable data were available, random-effects meta-analyses were performed. Risk ratios (RRs) were used for dichotomous outcomes and mean differences (MDs) for continuous outcomes, with 95% confidence intervals (CIs). Statistical heterogeneity was assessed using I^2^ statistic and T^2^. Outcomes reported by too few studies or with substantial differences in definitions or measurement methods were synthesized narratively rather than pooled. Meta-analysis was therefore restricted to outcomes for which studies provided sufficiently comparable interventions, comparators, outcome definitions, and numerical data. All analyses and generated figures were performed using Rstudio (2026.04.0+526) by meta package. A *p* value < 0.05 was considered significant in all analysis.

## 3. Results

### 3.1. Characteristics of Included Studies

**Figure 1:**
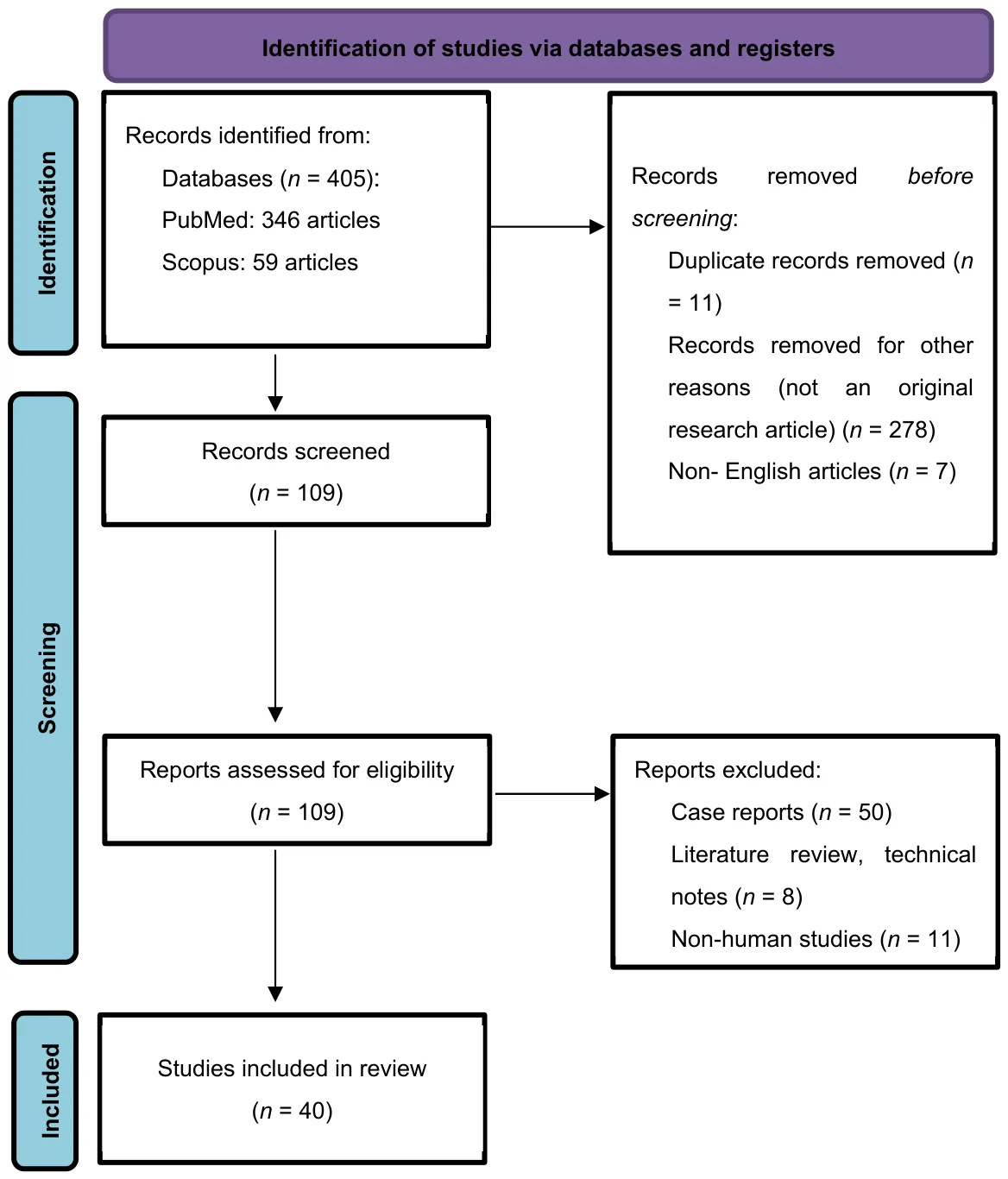
PRISMA flow diagram.

Table 1 below presents 3D-printing in spine surgery studies’ findings and characteristics. A total of 40 studies were included in this systematic review, Figure 1. A total of 28 out of the 40 (70.0%) reviewed studies were retrospective design studies [10,39,40,41,42,43,44,45,46,47,48,49,50,51,52,53,54,55,56,57,58,59,60,61,62,63,64,65]. There was a wide variation in sample size across studies, ranging from 7 to 192. The mean age for patients ranged from 10.8 to 77.59 years, while the standard deviation (SD) ranged between 1.4 and 17.9 years. Female patients were more common than males in 21 out of the 40 studies (52.5%) [10,28,39,41,42,43,46,49,50,51,52,55,56,61,62,65,66,67,68,69,70], male patients were more common than females in 18 out of the 40 (45.0%) [40,44,45,47,48,53,54,57,58,59,60,63,64,71,72,73,74,75], in 1 out of the 40 (2.5%) number of male and female patients were equal [76].

### 3.2. Outcomes of Interest in the Included Studies

All included studies were aimed at evaluating the accuracy, efficacy, precision, and safety of three-dimensional printing 3D-printing in diverse applications related to spine surgery, or at comparing it with another technique, or both.

### 3.3. Applications of 3D-Printing in the Field of Spine Surgery

In the field of spine surgery, the applications of 3D-printing included lumber procedure, deformity correction, precise screw placements, aid in treating cervical conditions and thoracolumbar trauma, and enhance precision, accuracy, safety, and efficacy in different spinal procedures through guided treatment and preoperative planning.

### 3.4. Precision and Outcomes of 3D-Printing in the Field of Spine Surgery

Numerous studies documented that 3D-printing increased precision in different spinal procedures [60,71,76]. In addition, nearly all studies reported achieving higher accuracy using this technique, and a significant decrease in misplacement-related complications was also reported [51]. All included studies documented favorable and superior outcomes using 3D-printing, including effectiveness, increased safety, enhanced quality of life, improved stability, and decline in the following: radiation exposure, blood loss, surgical time, implant subsidence rates, and postoperative complications. Zero-profile three-dimensional-printed artificial vertebral body (3DP-AVB) [48,72], individualized 3D-printing [46,73], and 3D-printing personalized model and drill guides [43,52] showed more favorable outcomes.

**Figure 2:**
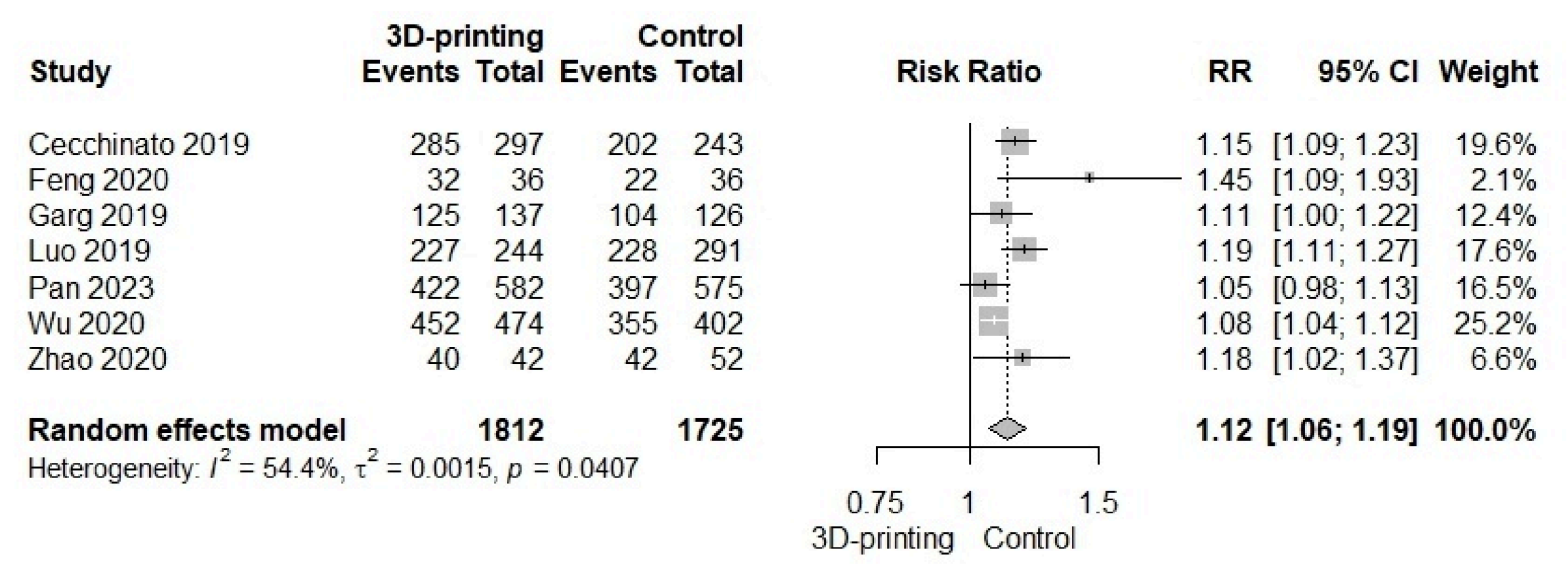
Forest plot comparing accuracy of 3D-printing against the control group. RR: risk ratio, CI: confidence interval [10,28,51,57,62,67,71].

Accuracy: Seven studies reporting screw placement accuracy were included in the meta-analysis. The random-effects model showed that 3D-printing-assisted instrumentation was associated with a significantly higher probability of achieving the defined accuracy outcome compared with conventional instrumentation (RR = 1.12, 95% CI: 1.06–1.19; *p* = 0.003). Moderate heterogeneity was observed among the included studies (I^2^ = 54.4%; Q = 13.15, *p* = 0.041). Although the direction of the effect generally favored 3D-printing-assisted instrumentation, the definitions of accuracy varied across studies and should therefore be considered when interpreting the pooled estimate, Figure 2.

**Figure 3:**
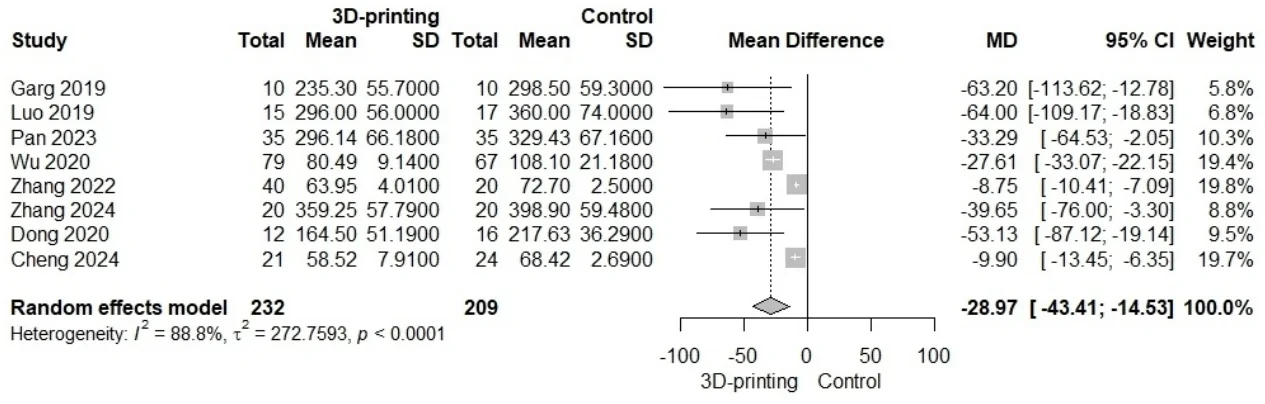
Forest plot comparing the surgical time of 3D-printing surgery against the control group. MD: mean difference, CI: confidence interval [10,40,41,51,57,60,67,75].

Surgical time: Eight studies were included in the quantitative synthesis. The random-effects meta-analysis demonstrated that three-dimensional printing was associated with a significantly shorter surgical time compared with conventional techniques (MD = −28.97 min, 95% CI −43.41 to −14.53; *p* < 0.001). However, substantial between-study heterogeneity was observed (I^2^ = 88.8%, τ^2^ = 272.76; Q = 62.30, *p* < 0.001), indicating considerable variability in the magnitude of the observed effect across studies. Therefore, the pooled estimate should be interpreted with caution, given differences among studies in patient populations, surgical procedures, and study characteristics, Figure 3.

**Figure 4:**
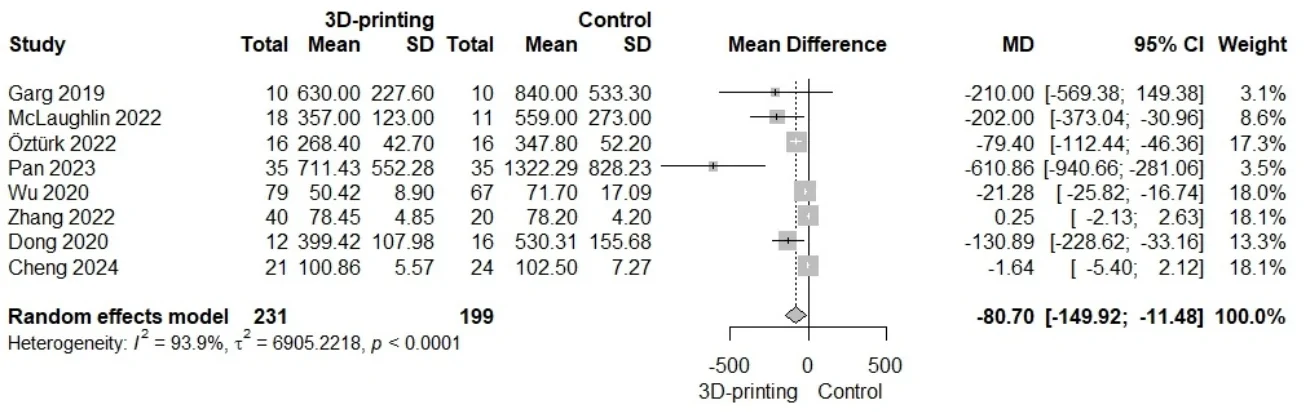
Forest plot comparing the blood loss of 3D-printing surgery against the control group. MD: mean difference, CI: confidence interval [10,40,41,50,51,57,68,75].

Blood loss: Eight studies were included in the quantitative synthesis. The random-effects meta-analysis showed that three-dimensional printing was associated with significantly lower intraoperative blood loss compared with conventional techniques (MD = −80.70 mL, 95% CI −149.92 to −11.48; *p* = 0.022). However, substantial between-study heterogeneity was observed (I^2^ = 93.9%, τ^2^ = 6905.22; Q = 115.38, *p* < 0.001), indicating considerable variability in the magnitude of the effect across studies. The pooled estimate should therefore be interpreted cautiously, as the observed differences in blood loss may reflect variation in patient populations, surgical procedures, baseline characteristics, and study methodologies, Figure 4.

### 3.5. Quality Assessment of Included Observational Studies

The Modified Newcastle–Ottawa scale was used to assess the quality of included non-randomized controlled studies. All included studies met the criteria of the first domain concerning study groups. A total of 17 out of 31 met the criteria of attrition (54.8%). All studies met the criteria of exposure measure and outcome measure. Investigators blinded domain was not applicable to the studies due to the non-interventional nature of the studies. The sixth domain concerning identifying confounders was met by only one study out of 31 studies (3.2%). The seventh domain concerning statistical adjustment was met by two studies out of 31 studies (6.5%). Funding source was mentioned clearly or disclosed by 17 out 31 studies (54.8%). The assessment considered study groups, attrition, exposure measurement, outcome measurement, investigator blinding, identification of potential confounders, statistical adjustment, and funding source. Each item was classified as Y (criteria met), N (criteria not met), NR (not reported), or N/A (not applicable), based on the information available in the original publication. For further details concerning quality assessment of included observational studies refer to Table 2.

### 3.6. Quality Assessment of Included Trials

Overall, there was low risk concerning deviations from intended interventions, missing outcome data, measurement of the outcome, and selection of reported results in all included trials. However, four out of nine studies (44.4%) showed high risk concerning their randomization process. In addition, two out of nine studies (22.2%) showed some concerns concerning their randomization process. Risk of bias assessment was performed using Cochrane Risk of Bias 2 (RoB 2) tool. The assessment covered five domains: bias arising from the randomization process, bias due to deviations from intended intervention, bias due missing outcome data, bias in measurement of the outcome, and bias in selection of the reported results. Each domain was judged as “low risk,” some concerns,” or “high risk” according to the signaling questions and decision algorithms of the RoB 2 tool [77]. Judgment was based on the information reported in the original studies. For further details concerning quality assessment of included trials refer to Table 3.

## 4. Discussion

This systematic review analyzed the available literature concerning applications, precision, and outcomes of 3D-printing in spine surgery. The main strength points for this systematic review are that it did not restrict its outcome measure to specific spine surgery indication. In addition, it provides an update systematic search for the available literature concerning applications, precision, and outcomes of 3D-printing in spine surgery.

This systematic review showed that in the field of spine surgery, the applications of 3D-printing included lumber procedure, deformity correction, precise screw placements, aid in treating cervical conditions and thoracolumbar trauma, and enhance precision, accuracy, safety, and efficacy in different spinal procedures through guided treatment and preoperative planning. Due to the extensive potential applications of 3D-printing in the field of spine surgery, there is a significant interest in the application of 3D-printing in other medical disciplines. Oral and maxillofacial surgery and orthopedic surgery were among the first to adopt this technology [78]. The utilization of this technology in spine surgery has been heightened by its advancement, as approximately 8% of the total literature on rapid prototyping pertains to its application in this field [37]. The potential applications of 3D-printing include the following: the development of patient-specific jigs or guides to optimize instrumentation placement, the development of patient-specific models for educational or preoperative planning purposes, the development of instrumentation or implants to meet the specific needs of a patient or goal, and the optimization of the structure of off-the-shelf implants [35,79]. The potential cost reductions associated with 3D-printing are derived from the ability to produce implants in which the manufacturing cost is primarily driven by the quantity of material printed, rather than the complexity of the design. As Mobbs et al. observe, the cost burden is transferred to the design of these devices, rather than the physical production, due to the necessity of skilled labor in the initial phases of the custom 3D device workflow [15]. While processes are constantly being enhanced, the time-intensive design processes necessary to produce custom implants limit their production [18].

This systematic review found that 3D-printing was associated with improved technical accuracy in several spinal procedures [60,71,76]. In addition, nearly all studies reported achieving higher accuracy using this technique, and a significant decrease in misplacement-related complications was also reported [51]. Overall, favorable outcomes were frequently reported with 3D-printing-assisted approaches; however, these findings should be interpreted cautiously given the methodological limitations and risk of bias identified in the included studies. Zero-profile three-dimensional-printed artificial vertebral body (3DP-AVB) [48,72], individualized 3D-printing [46,73], and 3D-printing personalized model and drill guides [43,52] showed more favorable outcomes. Spine surgery for complex deformities has been demonstrated to enhance surgical accuracy and intraoperative efficiency, while simultaneously reducing blood loss, through preoperative 3D modeling. Typically, 3D guides are employed to enhance the accuracy of screws in patients with complex anatomy or in regions where screw inaccuracy could result in substantial patient morbidity [18].

The observed benefits of 3D-printing may be explained by improved visualization of patient-specific anatomy and more detailed preoperative planning. Patient-specific models can help surgeons understand complex anatomical relationships before surgery, while customized guides can help translate the planned trajectory into the operative field and improve screw placement accuracy. These advantages maybe particularly relevant when anatomy is complex and distorted [32,80].

The findings suggest that 3D-printing may be particularly useful in complex spinal procedures, including deformity surgery, cervical and thoracolumbar pathology, trauma, and procedures requiring accurate instrumentations or reconstruction of irregular bony anatomy. In these settings, patient-specific models and guides may provide greater benefit than in routine procedures with predictable anatomy. However, the available evidence does not establish a unform benefit across all primal procedures [81].

The reported outcomes can be broadly categorized into technical, perioperative, and patient-centered clinical outcomes. Technical outcomes include screw placement accuracy and trajectory deviation, whereas perioperative outcomes include surgical time, blood loss, radiation exposure, and complications.

Considerable clinical and methodological heterogeneity was present across the included studies. The studies differed in design, patient age and characteristics, surgical procedures, indications, and applications of 3D-printing. Statistical heterogeneity was also observed in the quantitative syntheses, particularly for surgical time and blood loss. These differences may contribute to variation in the magnitude of the observed effects and limited the extent to which the pooled estimates can be generalized across different spinal surgical settings. Therefore, the findings should be interpreted as an overall synthesis of the available evidence rather than as a uniform effect applicable to all patient population and surgical procedures.

A systematic comparison of the present review with previous systematic reviews is provided in Supplementary Table S2, considering the search period, database searched, eligibility criteria, number and design of included studies, and outcome evaluation. In contrast to previous reviews on this subject, this review is more methodologically current and tightly focused, despite its narrower scope. Hajnal et al. and Araújo et al. clinical-trials-only update either cover a broader time span with less recent granularity or restrict inclusion to clinical trials only, excluding the retrospective case series that dominate this literature [82,83]. Wilcox et al. and Martínez Quiñones et al. both predate late developments in patient-specific implants and navigation guides [11,32]. Compared with previous reviews, the present review provides as explicit quality appraisal of the included studies and focuses specifically on the precision and clinical outcomes associated with 3D-prinitng application in spine surgery, while incorporating both randomized and observational evidence. In contrast to Hajnal et al., this review provides a synthesis that is more current, outcome-oriented, with a focus on technical accuracy, and that covers a broader range of spinal applications, including lumbar procedures, deformity correction, screw placement accuracy, and cervical and thoracolumbar trauma.

Future studies should include adequately powered randomized controlled trials directly comparing 3D-printing-assisted guides and implants with surgical techniques. Standardized definitions and outcomes measures for screw placement accuracy, surgical time, blood loss, complications, and patient-reported outcomes are also needed to improve comparability between studies. Multicenter studies with longer follow-up would further clarify whether improvements in technical outcomes translate into meaningful clinical benefits.

Although most studies showed low risk of bias in several domains, important methodological limitations were identified. Among the nine RCTs, four (44.4%) had a high risk of bias concerning the randomization process, while two (22.2%) had some concerns in this domain. Among the 31 observational studies, only one (3.2%) identified potential confounders and only two (6.5%) performed statistical adjustment for confounding. These limitations may affect the reliability of the observed associations and should be considered when interpreting the overall findings. Therefore, although favorable outcomes were frequently reported, the available evidence should be interpreted cautiously rather than as definitive evidence of superiority.

This review has several limitations. First the included studies showed considerable clinical and methodological heterogeneity in terms of study design, patient populations, surgical procedures, and 3D-printing applications, which may limit the generalizability of the findings. Second, several studies had important methodological limitations, including high or unclear risk of bias in the randomization process and limited identification and adjustment for potential confounders in observational studies. Third, quantitative synthesis was possible only for selected outcomes because many outcomes were reported by few studies or using different definitions and measurement approaches. Fourth, the relatively small number of studies available for some outcomes limited the ability perform reliable subgroup analyses. Additionally, restricting the review to English-language publications may have introduced language bias. Formal assessment of publication bias was not performed because the number of studies available for the quantitative syntheses was limited, making statistical assessment of publication bias unreliable. Therefore, the findings should be interpreted cautiously, and further well-designed prospective and randomized studies are needed provide more robust evidence. Finally, study selection and data extraction were performed by a single author, which may have introduced selection and extraction bias.

## 5. Conclusions

This systematic review and meta-analysis suggest that 3D-printing-assisted approaches may improve technical accuracy and reduce surgical time and intraoperative blood loss in spine surgery. However, substantial clinical and methodological heterogeneity and limitations in the quality of the available evidence warrant cautious interpretation of these findings. The applications of 3D-printing have evolved from preoperative planning and educational models to patient-specific guides and custom implants. Further well-designed prospective studies and randomized controlled trials are needed to confirm these findings and establish the clinical benefits of 3D-printing across different spinal procedures.

## Figures and Tables

**Table 1:** (**a**) 3D-printing applications in screw placement and spinal fixation. (**b**) 3D-printing applications in implants, cages, and spinal reconstruction. (**c**) 3D-printing applications in preoperative planning, surgical guidance, and deformity correction.

(**a**)						
Study	Study Design	Aim	Sample Size	M/F, Mean Age (Year)	Applications	3D-Printing Accuracy and Outcomes
Cecchinato et al. 2019 [28]	Open-label, parallel, single-center prospective RCT, 1:1 allocation	To evaluate the accuracy of pedicle screw insertion with 3DP guides (MySpine™) vs. FH insertion.	29 patients; MySpine™: 14; FH: 15	MySpine™: 2/12, 34 ± 15.3; FH: 1/14, 26 ± 17.2	Pedicle screw insertion with patient-specific 3DP guides based on low-dose CT in spinal deformity	Significantly decreased intraoperative radiation exposure, implantation-phase surgical time, and malpositioned screws. No difference in total surgical time. No increase in complications.
Chen et al. 2019 [66]	Prospective	To assess the accuracy and safety of PSP using 3DP drill templates in spinal deformity surgery.	10 patients	3/7, 18.9	Assistance in spinal deformity correction	High consistency, acceptability, and accuracy.
Dong et al. 2024 [42]	Retrospective	To evaluate the clinical effectiveness and safety of screw placement in type II old odontoid fractures using a 3DP NAV template vs. FH.	38 patients; NAV: 17; FH: 21	NAV: 9/8, 51.30 ± 13.20; FH: 7/14, 49.46 ± 11.92	Screw placement in type II old odontoid fractures	Significantly shorter surgical time. Enhanced accuracy of axis pedicle screw placement. No significant differences in hospital stay, postoperative drainage, blood loss, or atlas pedicle screw accuracy.
Feng et al. 2020 [71]	Randomized, single-blind controlled study	To assess and compare the precision and feasibility of cervical lateral mass screw insertion using 3DP templates vs. FH.	12 patients; 3DP: 6; FH: 6	3DP: 5/1, 57.67 ± 11.20; FH: 5/1, 17 ± 5.91	Cervical lateral mass screw fixation	Increased screw insertion accuracy and higher screw location precision. No significant difference in surgical time or clinical data.
Gao et al. 2017 [43]	Retrospective	To assess the effectiveness and feasibility of personalized 3DP models to facilitate treatment of CVJ abnormalities by posterior internal fixation.	44 patients; 3DP models made for 38	19/25, 36.5 ± 9.2	Posterior screw fixation for CVJ abnormalities	Significantly enhanced postoperative JOA scores, increased CMA, and decreased ADI. Feasible and practical; minimized intraoperative error and facilitated intraoperative guidance and preoperative simulation.
Garg et al. 2019 [10]	Retrospective comparative clinical study (quasi-randomized design)	To compare the safety and results of PSP in complex spinal deformity correction using 3DP vs. FH.	20 patients; non-3DP: 10; 3DP: 10	Non-3DP: 3/7, 15.5 (3.8); 3DP: 4/6, 16.6 (4.9)	Complex spinal deformities	Significantly enhanced screw placement accuracy and reduced surgical time. Lower fluoroscopy exposure. No significant difference in mean blood loss.
Liu et al. 2017 [49]	Retrospective	To assess outcomes and accuracy of PSP using a multilevel 3DP drill-guide template for rigid and severe scoliosis.	10 patients	4/6, 17.7	PSP in rigid and severe scoliosis	Multilevel 3DP templates were more effective and provided higher accuracy and better deformity correction than FH.
Luo et al. 2019 [67]	Prospective non-randomized controlled trial	To assess the accuracy, efficacy, and safety of pedicle screw placement in severe congenital scoliosis using 3DP and a pedicle guider vs. FH.	32 patients; guider: 15; control: 17	Guider: 6/9, 12 ± 3; control: 5/12, 14 ± 4	Pedicle screw placement in severe congenital scoliosis	3DP with the pedicle-guider technique provided higher accuracy and shortened surgical time.
McLaughlin et al. 2022 [50]	Retrospective cohort study	To explore the costs and advantages of AIS fusion using 3DP guides compared with FH.	29 patients; 3D: 18; FH: 11	3D: 5/13, 14.1 ± 1.4; FH: 6/5, 14 ± 1.8	Surgical management of AIS	Significantly increased costs and reduced screw placement time and intraoperative blood loss. No significant differences in other measures.
Öztürk et al. 2022 [68]	Randomized non-controlled study	To compare PSP accuracy and outcomes for AO spinal C-type injuries between conventional surgery and 3D model-assisted surgery.	32 patients; control: 16; experimental: 16	Control: 6/10, 37.2 ± 13.8; experimental: 8/8, 38.8 ± 13.4	Surgical planning in AO spine type-C fracture posterior long-segment fixation	Significantly shortened surgical time and reduced fluoroscopy exposure, medial axial encroachment, and blood loss.
Pijpker et al. 2021 [52]	Single-center retrospective case series	To evaluate the accuracy and safety of pedicle and lateral mass screw insertion in the upper thoracic and cervical regions using 3DP personalized drill guides.	15 patients	5/10, 53.1	Pedicle and lateral mass screw insertion	Accurate and safe.
Pu et al. 2018 [53]	Retrospective	To explore the accuracy, safety, and clinical outcomes of atlantoaxial PSP assisted by a modified 3DP NAV template.	17 patients	11/6, 43.3	Atlantoaxial PSP	Favorable clinical outcomes, enhanced patient safety, and enhanced screw placement accuracy.
Sugawara et al. 2018 [73]	Prospective clinical study	To assess the effectiveness of screw insertion into the thoracic and cervical regions using individualized SGTS.	103 patients	57/46; age range 15–85	Screw insertion into thoracic and cervical regions	Reduced radiation exposure, shortened surgical time, and enhanced PS insertion accuracy.
Thayaparan et al. 2020 [69]	Prospective	To document the efficacy, safety, and accuracy of SpineBox for MIS-TLIF.	129 patients	54/75, 59.2 ± 13.1	MIS-TLIF	Satisfactory implementation; PSP accuracy was 97.8%, with reoperation and total complication rates of 4.7% and 1.6%, respectively.
Tian et al. 2019 [54]	Retrospective	To compare the accuracy and safety of C2 pars or pedicle screw placement using a 3D-printed NAV template vs. C-arm NAV.	64 patients; Group A: 32; Group B: 32	Group A: 21/11, 47.2 ± 11.1; Group B: 19/13, 45.6 ± 10.4	C2 screw placement	C2 screw placement accuracy was higher with 3DP NAV template assistance. Clinical efficacy and safety were similar between groups.
Tu et al. 2021 [55]	Retrospective	To investigate clinical use of 3DP in surgical management of congenital scoliosis caused by hemivertebrae.	24 patients; 3DP: 11; conventional: 13	3DP: 2/9, 12.36 ± 2.62; conventional: 2/11, 12.23 ± 2.65	Surgical correction of congenital scoliosis due to hemivertebrae	Postoperative Cobb-angle correction was similar. Complication rate, screw placement accuracy, time per screw, transfusion, blood loss, and surgical time were significantly better in the 3DP group.
Wu et al. 2020 [57]	Retrospective	To assess the accuracy of PPS placement with a novel drill-guide template.	146 patients; guide: 79; conventional: 67	Guide: 46/33, 48.91 ± 7.34; conventional: 36/31, 47.55 ± 8.32	Assistance in PPS placement	Less radiation exposure, blood loss, and surgical time, with higher screw accuracy compared with traditional placement.
Wu et al. 2021 [58]	Retrospective	To examine the effectiveness and safety of a 3DP screw-rod auxiliary technique for unstable atlas fractures.	14 patients	11/3, 53.21 ± 14.81	Unstable atlas fractures	Effective with minimal complications.
Xu et al. 2017 [74]	Case series	To assess use of 3DP in preoperative planning of PSP for middle-upper thoracic trauma.	7 patients	5/2, 37.7	Preoperative PSP planning for middle-upper thoracic trauma	Efficacious and intuitive; decreased errors and enhanced screw placement accuracy.
Yuan et al. 2024 [59]	Retrospective	To assess the accuracy and safety of a 3DP double template for C1 and C2 PSP vs. FH.	60 cases; FH: 32; 3DP: 28	33/27; age range 42–69	C1 and C2 PSP	Facilitated secure C1 and C2 PSP. Significant difference in fluoroscopy times. No significant differences in surgical time or bleeding.
Zhang et al. 2022 [75]	Prospective, randomized by random-sequence method	To investigate the effectiveness of a 3DP percutaneous guide template with a lotus-style regulator in PPSF.	60 patients; traditional: 20; flat-end regulator: 20; pointed regulator: 20	Traditional: 9/11, 53.00 ± 2.51; flat-end: 10/10, 51.63 ± 2.72; pointed: 12/8, 52.00 ± 2.93	PPSF for thoracolumbar fractures	The 3DP guide with a pointed lotus-style regulator enhanced pedicle insertion accuracy and showed favorable results for other outcomes.
Zhang et al. 2020 [70]	Randomized controlled trial	To assess a 3DP template with porous polyoxymethylene thermoplastic regulator for PPSF in thoracolumbar fractures.	40 patients; 3DP: 20; control: 20	3DP: 5/15, 56.57 ± 5.50; control: 7/13, 57.33 ± 4.63	PPSF in thoracolumbar fractures	Significantly increased pedicle insertion success.
Zhao et al. 2021 [61]	Retrospective	To assess the accuracy and safety of a 3DP surgical guide template for S2AI screw placement.	27 patients	12/15, 57.56 ± 8.76	S2AI screw placement	Accurate and safe.
Zhao et al. 2020 [62]	Retrospective	To evaluate S2AI screw placement using a 3DP guide template vs. FH.	47 patients; guide: 21; FH: 26	Guide: 6/15, 56.9 ± 8.4; FH: 9/17, 58.6 ± 8.1	S2AI screw placement	Superior accuracy and safety.
Zhou et al. 2020 [64]	Retrospective	To present a 3DP TGT technique based on S2AI screw placement anatomical parameters in Asian patients and compare it with FH.	48 patients; FH: 28; TGT: 20	FH: 15/13, 63.7 ± 12.6; TGT: 11/9, 57.8 ± 9.5	S2AI screw placement	For S2AI placement in Asian patients, a 39° mediolateral angle and 30–35° CA were considered suitable. Both techniques were safe; TGT provided superior accuracy.
(**b**)						
Study	Study design	Aim	Sample size	M/F, Mean age (year)	Applications	3D-printing Accuracy and Outcomes
Alan et al. 2023 [39]	Retrospective observational cohort study	To compare reoperation and subsidence rates between PEEK and 3DP pTi following LLIF, including cost analysis and institutional propensity-score matching.	192 patients; before matching: PEEK 137 patients/212 levels, pTi 55 patients/97 levels; after matching: 97 lumbar levels/group	Before matching: PEEK 66/146, 62 ± 10.6; pTi 35/62, 64 ± 9.2. After matching: PEEK 23/74, 65 ± 7.9; pTi 35/62, 64 ± 9.2	LLIF	Significantly less subsidence. No statistical difference in revision rates. More cost-effective when at least $1185.9 cheaper than PEEK.
Cheng et al. 2024 [40]	Retrospective	To assess the efficacy of titanium-alloy trabecular bone 3DP-AVB for cervical OPLL.	45 patients; study: 21; control: 24	Study: 12/9, 47.8 ± 5.2; control: 13/11, 48.1 ± 7.9	Treatment of cervical OPLL	Reduced surgical time, implant subsidence, and postoperative complications. Superior vertebral reconstruction.
Dong et al. 2020 [41]	Retrospective	To compare radiographic and clinical outcomes of 3DP-AVB vs. TMC in KD with neurological deficits.	28 patients; 3DP-AVB: 12; TMC: 16	3DP-AVB: 3/9, 70.75 ± 7.96; TMC: 5/11, 73.44 ± 8.06	Treatment of KD with neurological deficits	Reduced surgical time, cage subsidence, loss of kyphosis correction, and blood loss. More reasonable long-term effectiveness for daily life function, back pain, and preservation of fused-segment height.
Hu et al. 2024 [44]	Single-arm retrospective cohort study	To investigate short-term consequences of a 3DP stand-alone AVB in ACCF.	11 patients	7/4, 57.2 ± 10.7	Surgical procedure of ACCF	Significant improvement in cervical lordosis, adequate fusion rate, and relatively low complication rate.
Li, Li, Yan, et al. 2024 [48]	Retrospective	To assess the efficacy and safety of self-stabilizing zero-profile 3DP-AVB in ACCF for CSM.	37 patients; treatment: 15; control: 22	Treatment: 10/5, 50.7 ± 17.0; control: 14/8, 54.7 ± 14.1	ACCF for CSM	Compared with 3DP-AVB, self-stabilizing zero-profile 3DP-AVB provided comparable efficacy, less postoperative dysphagia, and reduced surgical time.
Li, Li, Gu, et al. 2024 [72]	Prospective randomized controlled trial	To compare a 3DP self-stable zero-profile AVB vs. titanium mesh for ACCF in cervical OPLL.	41 patients; zero-notch: 21; titanium mesh: 20	Zero-notch: 16/5, 48.0 ± 12.7; titanium mesh: 14/6, 49.8 ± 10.2	ACCF for cervical OPLL	Compared with TMC, 3DP self-stable zero-profile AVB provided better surgical clinical effectiveness and shorter surgical time.
Wang et al. 2024 [56]	Retrospective cohort study	To compare radiological and clinical postoperative outcomes of 3DP Ti6Al4V vs. PEEK interbody fusion cages in posterior lumbar fusion.	91 patients; 3DP: 39; PEEK: 52	3DP: 16/23, 61.28 ± 9.06; PEEK: 18/34, 61.52 ± 6.63	Interbody fusion cages in posterior lumbar fusion	Good clinical outcomes in both groups. Fusion rates were comparable (89.13% vs. 90.91%). Collapse rates were 6.5% vs. 4.5%. No significant difference in cage displacement.
Zhou et al. 2023 [63]	Retrospective case study	To evaluate 3DP-AVB vs. TMC for reducing prosthesis subsidence in cervical chordoma surgery.	24 patients; 3DP-AVB: 9; TMC: 15	3DP-AVB: 4/5, 45.56 ± 10.03; TMC: 9/6, 50.27 ± 14.41	Surgical management of cervical chordoma	Significantly reduced prosthesis subsidence and enhanced stability.
(**c**)						
Study	Study design	Aim	Sample size	M/F, Mean age (year)	Applications	3D-printing accuracy and Outcomes
Ding et al. 2023 [76]	Prospective	To document use of a 3DP guidance template and tailored preoperative digital planning in correcting complicated and severe ASD.	8 patients	4/4, 33.75	Treatment of severe and complex ASD	Effective; increased precision, safety, and accuracy.
Jiang et al. 2018 [45]	Retrospective	To investigate computer-NAV-assisted minimally invasive DLIF for lumbar tuberculosis.	33 patients; NAV: 18; non-NAV: 15	NAV: 10/8, 54.1 ± 17.5; non-NAV: 7/8, 57.6 ± 17.9	Assistance in minimally invasive DLIF for lumbar tuberculosis	Significantly decreased intraoperative radiation exposure time. No significant differences in other outcomes.
Karlin et al. 2017 [46]	Retrospective	To assess individualized 3DP models as an assisting tool for spinal deformity correction in children with myelomeningocele.	17 patients; 3D models: 7; non-models: 10	3D models: 3/4, 10.8; non-models: 4/6, 13.4	Treatment of spinal deformity in children with myelomeningocele	Comparable or superior surgical efficiency regarding deformity correction, blood loss, and intraoperative fluoroscopy time. Facilitated individualized spinal tools and significantly enhanced understanding of complex anatomy.
Lador et al. 2020 [47]	Retrospective	To examine 3DP utilization in complicated spine surgical procedures.	7 patients	5/2, 32.3	Complex spine surgeries	Enhanced reconstruction, diminished surgical complexity, and improved preoperative planning.
Pan et al. 2023 [51]	Retrospective case–control study	To assess the significance of 3DP spine models in severe spinal deformity surgical correction.	70 patients; 3DP: 35; control: 35	3DP: 10/25, 25.37 ± 11.06; control: 9/26, 26.14 ± 8.73	Severe spine deformity correction surgery	Enhanced efficiency and safety, higher screw placement accuracy, and shorter surgical time. Blood loss and screw-misplacement-related complications were significantly lower.
Zhang et al. 2024 [60]	Single-center retrospective comparative cohort study	To assess the precision, safety, and effectiveness of managing complicated and severe ASD requiring 3-CO using a 3DP osteotomy guide template vs. FH osteotomy.	40 patients; 20/group	Template: 12/8, 36.70 ± 12.44; non-template: 12/8, 36.25 ± 11.83	Treatment of complicated and severe ASD requiring 3-CO	Shortened surgical time, improved execution precision, and reduced risk of postoperative neurological deficit.

M, male; F, female; 3DP, three-dimensional printing; PEEK, polyetheretherketone; pTi, porous titanium; LLIF, lateral lumbar interbody fusion; FH, freehand; PSP, pedicle screw placement; OPLL, ossification of posterior longitudinal ligament; 3DP-AVB, three-dimensional-printed artificial vertebral body; TMC, titanium mesh cage; KD, Kümmell’s disease; CVJ, craniovertebral junction; NAV, navigation; AVB, artificial vertebral body; ACCF; anterior cervical corpectomy and fusion; JOA, Japanese Orthopedic Association; CMA, cervicomedullary angle; ADI, atlanto-dens interval; DLIF, direct lateral interbody fusion; PVP, percutaneous vertebroplasty; CSM, cervical spondylotic myelopathy; AIS, adolescent idiopathic scoliosis; SGTS, screw guide template system; MIS TLIF, minimally invasive transforaminal lumbar interbody fusion; PPS, percutaneous pedicle screw; PPSF percutaneous pedicle screw fixation; TLSO, thoracolumbosacral orthoses; ASD, adult spinal deformity; 3-CO, three-column osteotomy; S2AI, S2 alar-iliac; TGT, template guided technique; CA, cephalocaudal angle.

**Table 2:** Modified Newcastle–Ottawa scale for appraisal of non-randomized controlled studies.

Study	Study Groups	Attrition	Exposure Measure	Outcome Measure	Investigators Blinded	Confounders Identified	Statistical Adjustment	Funding Source
Alan et al. 2023 [39]	Y	NR	Y	Y	N/A	Y	Y	NR
Chen et al. 2019 [66]	Y	NR	Y	Y	N/A	N	N	NR
Cheng et al. 2024 [40]	Y	NR	Y	Y	N/A	N	N	NR
Ding et al. 2023 [76]	Y	N	Y	Y	N/A	N	N	Y
Dong et al. 2020 [41]	Y	Y	Y	Y	N/A	N	N	Y
Dong et al. 2024 [42]	Y	Y	Y	Y	N/A	N	N	Y
Gao et al. 2017 [43]	Y	Y	Y	Y	N/A	N	N	Y
Hu et al. 2024 [44]	Y	Y	Y	Y	N/A	N	N	Y
Jiang et al. 2018 [45]	Y	Y	Y	Y	N/A	N	N	Y
Jiang et al. 2024 [65]	Y	Y	Y	Y	N/A	N	N	Y
Karlin et al. 2017 [46]	Y	NR	Y	Y	N/A	N	N	NR
Lador et al. 2020 [47]	Y	NR	Y	Y	N/A	N	N	NR
Li, Li, Yan et al. 2024 [48]	Y	Y	Y	Y	N/A	N	N	NR
Li, Li, Gu et al. 2024 [72]	Y	Y	Y	Y	N/A	N	N	NR
Liu et al. 2017 [49]	Y	Y	Y	Y	N/A	N	N	NR
McLaughlin et al. 2022 [50]	Y	Y	Y	Y	N/A	N	Y	Y
Pan et al. 2023 [51]	Y	N	Y	Y	N/A	N	N	Y
Pijpker et al. 2021 [52]	Y	N	Y	Y	N/A	N	N	Y
Thayaparan et al. 2020 [69]	Y	Y	Y	Y	N/A	N	N	NR
Tian et al. 2019 [54]	Y	Y	Y	Y	N/A	N	N	NR
Tu et al. 2021 [55]	Y	Y	Y	Y	N/A	N	N	NR
Wang et al. 2024 [56]	Y	Y	Y	Y	N/A	N	N	Y
Wu et al. 2020 [57]	Y	N	Y	Y	N/A	N	N	Y
Wu et al. 2021 [58]	Y	N	Y	Y	N/A	N	N	Y
Xu et al. 2017 [74]	Y	Y	Y	Y	N/A	N	N	Y
Yuan et al. 2024 [59]	Y	N	Y	Y	N/A	N	N	NR
Zhang et al. 2024 [60]	Y	Y	Y	Y	N/A	N	N	Y
Zhao et al. 2021 [61]	Y	N	Y	Y	N/A	N	N	NR
Zhao et al. 2020 [62]	Y	Y	Y	Y	N/A	N	N	Y
Zhou et al. 2023 [63]	Y	N	Y	Y	N/A	N	N	NR
Zhou et al. 2020 [64]	Y	N	Y	Y	N/A	N	N	Y

**Note:** Y = criteria met; N = criteria not met; NR = not reported; N/A = not applicable.

**Table 3:** Quality assessment of included trials.

Study	Randomization Process	Deviations from Intended Interventions	Missing Outcome Data	Measurement of the Outcome	Selection of Reported Results
Cecchinato et al. 2019 [28]	Low risk	Low risk	Low risk	Low risk	Low risk
Feng et al. 2020 [71]	High risk	Low risk	Low risk	Low risk	Low risk
Garg et al. 2019 [10]	Low risk	Low risk	Low risk	Low risk	Low risk
Luo et al. 2019 [67]	High risk	Low risk	Low risk	Low risk	Low risk
Öztürk et al. 2022 [68]	Some concerns	Low risk	Low risk	Low risk	Low risk
Pu et al. 2018 [53]	High risk	Low risk	Low risk	Low risk	Low risk
Sugawara et al. 2018 [73]	High risk	Low risk	Low risk	Low risk	Low risk
Zhang et al. 2022 [75]	Low risk	Low risk	Low risk	Low risk	Low risk
Zhang et al. 2020 [70]	Some concerns	Low risk	Low risk	Low risk	Low risk

## Data Availability

No new data were created or analyzed in this study. Data sharing is not applicable to this article.

## Supplementary Materials

The following supporting information can be downloaded at: https://www.mdpi.com/article/10.3390/healthcare14193344/s1, Supplementary Table S1. Summary of data extracted from the original articles for meta-analysis; Supplementary Table S2. Comparison of the present systematic review with previous reviews of 3D printing in spine surgery.
