# Supplementary material for: A Systematic Review of 3D-Printing in Spine Surgery: Applications, Accuracy, and Outcomes
Source: Healthcare (Basel). 2026 Oct 8;14(19):3344. doi: 10.3390/healthcare14193344 (PMC13664814; doi:10.3390/healthcare14193344)
Supplement: Supplementary file 1 [file healthcare-14-03344-s001.zip › healthcare-4531228-supplementary.pdf]

Supplementary Table S1. Summary of data extracted from the original articles for meta-analysis

Study	Outcome	3DP Mean	3DP SD	3DP N	Control Mean	Control SD	Control N	3DP Events	3DP Total	Control Events	Control Total
Cecchinato 2019	Accuracy							285	297	202	243
Feng 2020	Accuracy							32	36	22	36
Garg 2019	Accuracy							125	137	104	126
Garg 2019	Surgical time	235.3	55.7	10	298.5	59.3	10				
Garg 2019	Blood loss	630	227.6	10	840	533.3	10				
Luo 2019	Accuracy							227	244	228	291
Luo 2019	Surgical time	296	56	15	360	74	17				
McLaughlin 2022	Blood loss	357	123	18	559	273	11				
Öztürk 2022	Instrumentation time	61.9	4.7	16	75.5	11	16				
Öztürk 2022	Blood loss	268.4	42.7	16	347.8	52.2	16				
Pan 2023	Surgical time	296.14	66.18	35	329.43	67.16	35				
Pan 2023	Blood loss	711.43	552.28	35	1322.29	828.23	35				
Pan 2023	Accuracy							422	582	397	575
Wu 2020	Surgical time	80.49	9.14	79	108.1	21.18	67				
Wu 2020	Blood loss	50.42	8.9	79	71.7	17.09	67				
Wu 2020	Accuracy							452	474	355	402
Zhang 2022	Surgical time	63.95	4.01	40	72.7	2.5	20				
Zhang 2022	Blood loss	78.45	4.85	40	78.2	4.2	20				
Zhang 2024	Surgical time	359.25	57.79	20	398.9	59.48	20				
Alan 2023	Subsidence							8	97	26	97
Dong 2020	Subsidence							5	12	14	16
Dong 2020	Surgical time	164.5	51.19	12	217.63	36.29	16				

Dong 2020	Blood loss	399.42	107.98	12	530.31	155.68	16				
Cheng 2024	Surgical time	58.52	7.91	21	68.42	2.69	24				
Cheng 2024	Blood loss	100.86	5.57	21	102.5	7.27	24				
Cheng 2024	Complications							2	21	9	24
Wang 2024	Fusion							41	46	60	66
Wang 2024	Collapse							3	46	3	66
Zhou 2020	Iliac cortex penetration							1	40	10	56
Zhao 2020	Accuracy							40	42	42	52

Supplementary Table S2. Comparison of the present systematic review with previous reviews of 3D printing in spine surgery

**Review**	**Search period**	**Databases**	**No. studies**	**Study designs**	**Outcomes**
**Wilcox et al. 2017 [11]**	From database inception to March 2017	Ovid MEDLINE, PubMed, Cochrane CENTRAL, Cochrane Database of Systematic Reviews, ACP Journal Club, DARE	**54**	Mixed clinical evidence, including case reports/series and comparative studies	Surgical planning, intraoperative guides, customized prostheses/implants, surgical accuracy, operative time, patient outcomes, costs and technical considerations
**Martínez Quiñones et al. 2024 [81]**	Previous 10 years	PubMed, Embase, Cochrane Database of Systematic Reviews, Google	**38**	Mixed clinical studies	Surgical planning, medical education, doctor–patient communication, navigation templates, spinal implants,

		Scholar, OpenGrey			accuracy, precision, safety and clinical utility
**Araújo et al. 2024 [80]**	2014–March 2024	PubMed	**10**	Clinical trials	Personalized guides, surgical planning, patient education, resident education, surgical accuracy and clinical applications
**Hajnal et al. 2025 [79]**	January 1999–December 2022	PubMed, Web of Science, Scopus, Embase	**105**	44 retrospective, 50 prospective, 11 case series	Clinical outcomes, pedicle-screw accuracy, operative time, blood loss, complications, cost, printing materials/technology, printed object type and clinical application
**Present review**	Inception to November 7, 2024	PubMed, Scopus, Cochrane	**40**	9 RCTs + 31 observational studies	Accuracy, operative time, blood loss, complications, clinical outcomes, fusion/reconstruction outcomes, and other procedure-specific outcomes
